# Failure Analysis of a C-276 Alloy Pipe in a Controlled Decomposition Reactor

**DOI:** 10.3390/ma15072483

**Published:** 2022-03-28

**Authors:** Tai-Cheng Chen, Gui-Lin Yue, Wu Kai, Ren-Kae Shiue, Leu-Wen Tsay

**Affiliations:** 1 Department of Materials Science and Engineering, National Taiwan University, Taipei 10617, Taiwan; tcchen@iner.gov.tw (T.-C.C.); rkshiue@ntu.edu.tw (R.-K.S.); 2 Nuclear Fuels and Materials Division, Institute of Nuclear Energy Research, Taoyuan 32546, Taiwan; 3 Engineering Training Centre, Jiangsu Ocean University, Lianyungang 222005, China; yueglin@163.com; 4 Doctoral Degree Program in Ocean Engineering Technology, National Taiwan Ocean University, Keelung 20224, Taiwan; 5 Department of Optoelectronics and Materials Technology, National Taiwan Ocean University, Keelung 20224, Taiwan; wkai@mail.ntou.edu.tw

**Keywords:** C-276 alloy, failure analysis, intermetallic compound, δ phase, μ phase, intergranular fracture

## Abstract

Failure analysis was carried out on a ruptured C-276 pipe heated externally at 1050 °C, which had been used for a few months in a controlled decomposition reactor (CDR) system. To catch the decomposed perfluorinated compounds (PFCs, e.g., CF_4_, SF_6_, NF_3_, C_3_F_8_ and C_4_F_8_) present in the exhaust gas, the C-276 reactor was periodically purged with water mist, which caused a temperature gradient from the external to the inner surface of the pipe. The precipitation of large amounts of intermetallic compounds along the grain boundaries were found to be corroded preferentially. The internal surface of the used pipe was covered with many fine cracks. The corrosion and cracking of grain boundary precipitates accounted for the short service life of the C-276 pipe. Compositional measurements by electron probe micro-analyzer (EPMA) and phase identification by electron backscatter diffraction (EBSD) confirmed the presence of δ and μ phases in the ruptured pipe. The coarse intergranular precipitates were the δ phase (Mo_7_Ni_7_), which were enriched in Mo and Cr. Moreover, the fine precipitates dispersed intergranularly and intragranularly were the μ phase (Mo_6_Ni_7_), which were abundant in Mo and W. The numerous precipitates present in the matrix and along the grain boundaries were responsible for an obvious loss in the strength and ductility of the used C-276 pipe.

## 1. Introduction

Hastelloy C (Ni-based) alloys are used extensively in the chemical and aerospace industries and in marine applications due to their excellent resistance to harsh corrosion and high temperature oxidation. Hastelloy C-276, a typical Ni-Cr-Mo-W alloy [1], is known for its promising mechanical and physical properties. Raghavan et al. [2] pointed out that different precipitates (μ phase, M_6_C carbide and P phase) will form in C-276 alloy after aging in the temperature range of 650 to 900 °C; μ and P phases are both enriched in Mo and W. Precipitation of the Mo-enriched μ phase is known to produce a moderate loss of room-temperature tensile ductility [3] but results in considerable degradation of impact toughness [3,4]. Impact fracture of an embrittled C-276 alloy after aging at 850 °C for 120 h has been identified as complete intergranular fracture [4]. In addition, the μ phase also has a detrimental effect on the isothermal oxidation behavior of the Ni-Cr-Mo-W alloy [5]. The selective dissolution of μ particles and the presence of defects at the interface result in an increased oxidation rate of the alloy at high temperature [5]. A Mo and W depletion zone can form in the area near the grain boundary precipitates, and this zone is the main cause of the intergranular attack [6]. Moreover, high concentrations of Mo and W in the Ni-Cr-Mo alloy lead to the formation of a topologically close-packed (TCP) phase during solidification; the P phase forms at relatively higher temperature than the μ phase [5].

Nowadays, perfluorinated compounds (PFCs, e.g., CF_4_, SF_6_, NF_3_, C_3_F_8_ and C_4_F_8_) are extensively used by semi-conductor companies [7,8,9]. Several hundred tons of PFCs are applied each year in the foundries for etching Si wafers by chemical vapor deposition. Nitrogen trifluoride (NF_3_) is known to be an extremely strong greenhouse gas for it has a global warming potential thousands of times greater than that of CO_2_ [10,11,12]. Unfortunately, the use of nitrogen trifluoride as a plasma etchant of silicon wafers is increasing. To reduce the emission of PFCs in exhaust gas, a controlled decomposition reactor (CDR) system heated externally by an electrical heater at 1050 °C is applied to decompose the PFCs. The pipe is periodically purged with water mist to catch decomposed NF_3_ gas and other harmful species. Failure analysis was carried out on a failed C-276 pipe which ruptured after just a few months of service. A detailed investigation, including visual inspection, microstructural evaluation and phase identification, was performed to determine the causes of the fast failure of the C-276 pipe. In a prior study, the wear resistance and the hardness of Inconel 625 alloy could be increased by reinforcing with TiC particles with no harm to corrosion resistance, if less than 25% TiC was added [13]. Based on the results of this work, the surface modification of the C-276 alloy or the search for an alternative alloy for CDR system was planned to extend the service life.

## 2. Material and Experimental Procedures

C-276 pipe having an outer diameter of 170 mm and a wall thickness of 4 mm was used in a CDR system. The chemical composition of the C-276 alloy in wt.% was: 0.003 C, 0.50 Mn, 0.04 Si, 0.008 P, 0.001 S, 0.1 Co, 16.2 Cr, 15.7 Mo, 3.2 W, 6.7 Fe and the balance Ni. The as-received C-276 plate had been solution-annealed at 1170 °C in a mill. The as-received C-276 plate was rolled into a round pipe and then butt-welded by plasma arc welding process in the keyhole mode.

The microstructures of the ruptured pipe were investigated after it was cut into small samples, which were then subjected to metallographic preparations. A JSM-7100F field emission scanning electron microscope (SEM, JEOL, Tokyo, Japan) was used to examine the changes in the microstructure in the ruptured C-276 pipe. The chemical compositions of the precipitates were determined with a JXA-8200 electron probe micro-analyzer (EPMA, JEOL, Tokyo, Japan). The reactive products that had formed on the inner and outer surfaces of the ruptured C-276 pipe were investigated by D2 Phaser X-ray diffraction (XRD, Bruker, Karlsruhe, Germany) under Cu Kα radiation. The precipitates at the grain boundaries of the ruptured pipe were cut with a Helios NanoLab 600i focused ion beam (FIB, FEI, Hillsboro, OR, USA) for further examination. The tensile properties of the solution-annealed plate and samples cut from the ruptured pipe were determined at a strain rate of 6 × 10^−4^/s at room temperature using an 810 material testing system (MTS, Eden Prairie, MN, USA). The tensile test follows the ASTM E8M specification. To evaluate the degradation in strength/ductility of the used pipe, some tensile specimens were cut from the ruptured pipe in two different zones located at distinct distances away from the broken zone. Because of non-uniform thickness and geometry constriction of the round pipe, tensile test samples cut from the ruptured pipe showed a slight curved profile. To reduce the deviation in tensile properties of the tested sample, the sample thickness was measured at five locations within the 25 mm gage length before tensile tests. The microstructures of the precipitates were inspected in detail with a Tecnai G2 transmission electron microscope (TEM, JEOL, Tokyo, Japan). The samples for TEM examination were cut from the ruptured pipe by FIB. The specimens were also examined with a SEM equipped with a NordlysMax^2^ electron backscatter diffraction (EBSD, Oxford Instruments, Abingdon, UK) detector to identify the phases present in the specimens.

## 3. Results

### 3.1. Visual Inspection

**Figure 1:**
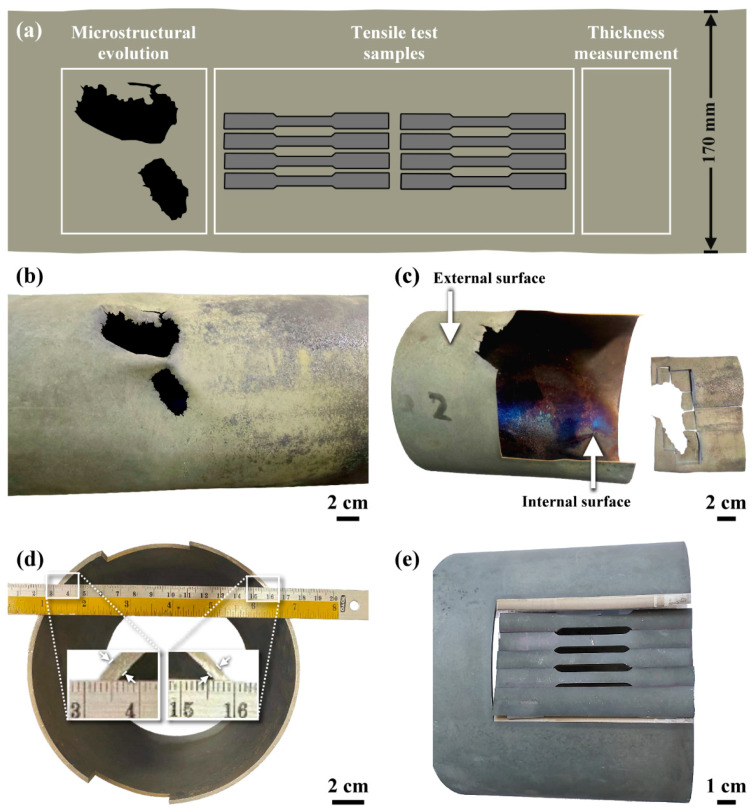
(**a**) Schematic diagram showing the investigated samples cut from the ruptured pipe, (**b**) the macro-appearance of the ruptured C-276 pipe with the caved-in holes on the pipe, (**c**) the samples sectioned by wire-cutting for microstructural examination, (**d**) the uneven thickness of the ruptured pipe, (**e**) the tensile samples cut from the failed pipe.

Figure 1 shows the schematic diagram of the investigated samples cut from the ruptured C-276 pipe and the macro-view of various samples. The C-276 pipe had failed in a much shorter time than the expected service life. As mentioned previously, the C-276 pipe was constructed by plasma-arc welding of the rolled plate in the keyhole mode. The fusion zone and heat-affected zone are known to be the weak sites of the C-276 weld [14,15,16]. To prevent the premature failure of the pipe, the weld metal was installed on the low temperature side of the CDR system. By contrast, the C-276 pipe without the weld seam was located on the high temperature side, which was heated by an external heater to a constant 1050 °C. The inlet exhaust gas containing NF_3_, NH_3_, SiH_4_, SiO_2_ was decomposed in the pipe at elevated temperature and pressure lower than atmospheric pressure. To catch the decomposed harmful species, the pipe was purged periodically with water mist, which caused a temperature gradient from the external to the inner surface of the pipe. It was expected that HNO_3_ and HF would be the reaction products of the decomposed exhausted gas. Thus, the inner side of the pipe would be corroded severely by the reaction products of the decomposed exhausted gas.

As shown in Figure 1b,c, the macro-appearance of the ruptured C-276 pipe showed that the pipe caved in and cracked because the reactor was operating under low pressure relative to the external atmosphere. The color of the external and internal surfaces of the ruptured pipe was dark green and dark gray, respectively. The colors of the products formed on the external and inner surfaces of the ruptured pipe could also be used to identify the specific species on the surfaces. It was observed that the edges of the holes of the C-276 pipe had been etched to less than 0.5 mm thickness. Moreover, many fine cracks around the hole extended into the surrounding substrate. It was noticed that the inner side of the C-276 pipe was etched much more obviously than the outer side of the pipe. The uneven temperature distribution around the pipe also led to a non-uniform reduction in thickness (Figure 1d). Besides, the external surface being heated to 1050 °C and water mist sprayed into the pipe contributed to high temperature oxidation of the pipe, particularly that of the external surface. Under such circumstances, the C-276 pipe was experiencing very harsh conditions.

### 3.2. Mechanical Properties Evaluation

The tensile properties of the as-received C-276 plate were as follows: yield strength of 370 MPa, ultimate tensile strength of 782 MPa and 56% elongation. As shown in Figure 1e, the tensile samples were cut from the ruptured pipe at different distances from the broken holes. As mentioned previously, the pipe was thinned non-uniformly to different thicknesses at different locations. After wire-cutting, the measured thickness near to the fracture site of the tested sample was used to calculate the sample’s tensile strength. The results of the tensile test and the sample condition are listed in Table 1. A short term service caused an obvious degradation in tensile properties of the used C-276 pipe, especially for the sample with thinner thickness. It revealed an over 23% loss in strength and 42% loss in ductility for the tensile samples cut from the pipe of about 2.5 mm in thickness. In the case of the samples with a thickness less than 0.9 mm, a further loss in strength and nil ductility of the specimens were obtained. A marked decrease in the tensile properties of the specimens could be related to the degraded microstructure and the many microcracks in the pipe. In fact, fine surface cracks were present mainly along the grain boundaries all over the internal surface of the used C-276 pipe, which would be examined and confirmed later in the text.

### 3.3. Microstructural Observation

**Figure 2:**
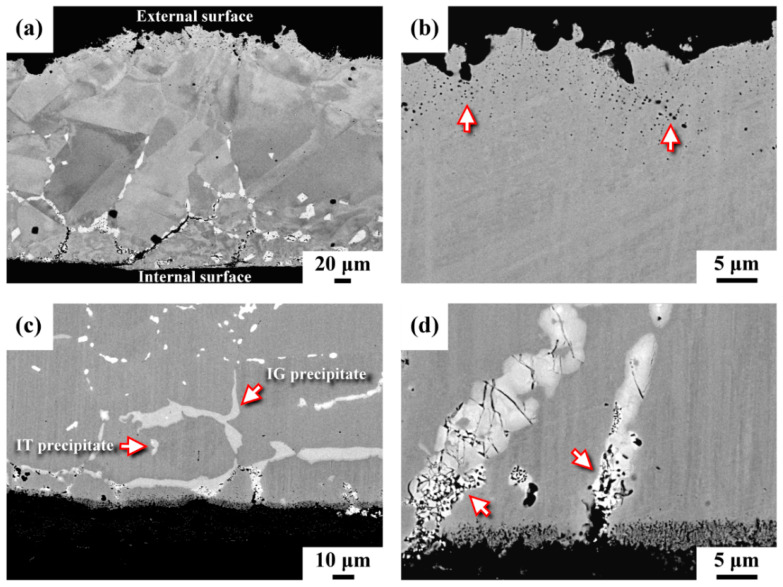
The microstructures of the samples cut from the ruptured pipe in the cross-sectional view: (**a**) the thinned sample beside the rupture, (**b**) the microstructure around the external surface, (**c**) the microstructure around the inner surface, (**d**) the etched inner surface and the cracking of coarse precipitates.

Figure 2 presents the microstructures of the ruptured pipe in cross-sectional view. It was noticed that the thickness of the pipe near the rupture had been thinned to less than 300 μm, as shown in Figure 2a. The external surface in the irregular profile was covered by thin scales. At higher magnification, many fine pinholes, indicated by the arrows, were found just beneath the outer surface (Figure 2b). Surface scales cracked and detached from the outer surface of the pipe during service. Moreover, no precipitates were found in the region near the external surface of the pipe, as shown in Figure 2a,b. In contrast, numerous precipitates in the back scattered electron (BSE) image were observed on the inner side of the pipe (Figure 2c). Overall, the intergranular (IG) precipitates were much coarser and greater in amount than the intragranular (IT) precipitates. It was noticed that two kinds of precipitates in gray and bright images were present in the investigated samples (Figure 2c). The results also indicated that the IG precipitates were corroded preferentially (as indicated by the arrow in Figure 2d), resulting in the formation of fine pores within the coarse precipitates. The temperature gradient between the outer and inner regions of the pipe inevitably induced thermal stress, which was responsible for severe cracking of the IG precipitates (Figure 2d). Moreover, the reaction products of decomposed perfluorinated compounds and oxidizing environment in the pipe led to an extensive, severe corrosion attack of the matrix, which resulted in forming an etched porous layer on the inner surface of the pipe (Figure 2d). It was deduced that thermal stress assisted the cracking of the grain boundary precipitates. Furthermore, the coalescence of micro-pores into micro-cracks and the linkage of micro-cracks resulted in the fast crack growth along the grain boundaries of the pipe. Furthermore, the combination of thermal stress and the corrosion attack caused spalling of the corroded segments, which was responsible for the rapid thinning in the wall of the C-276 pipe.

### 3.4. Chemical Composition Analysis

**Figure 3:**
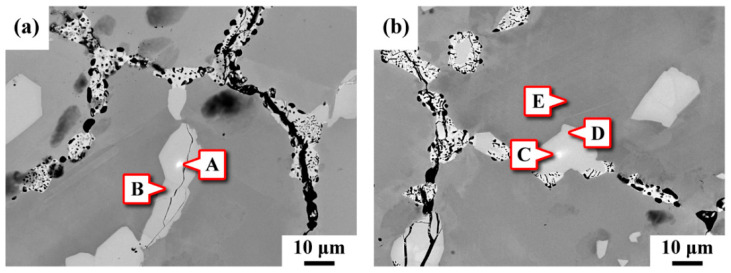
The SEM-BSE photos showing the precipitates in the C-276 pipe and the corresponding chemical compositions determined by EPMA at the specific sites are listed in Table 2: (**a**) the intragranular and (**b**) the intergranular precipitates.

Figure 3 shows the SEM morphologies of the precipitates present in the C-276 pipe in BSE images and the corresponding chemical compositions determined by EPMA at the specific sites are listed in Table 2. The main elements (in wt.%) of the substrate were 16.2 Cr, 15.7 Mo, 3.2 W, 6.7 Fe and the balance Ni. The results indicated that the IT and IG precipitates were all rich in Mo (sites A–D). It was noted that two kinds of precipitates distinguished by the brightness in the BSE images were present in the samples. Some bright spots (sites A and C) were embedded in the coarse precipitates (gray). As listed in Table 2, it was obvious that the bright fine particles were highly enriched in Mo and W but lean in Cr, relative to the substrate. Compared with the W-rich precipitates, the coarse precipitates were rich in Cr (sites B and D). It was deduced that at least two different kinds of precipitates were present in the ruptured pipe. Overall, the C and O contents in the detected precipitates were very low. It was obvious that the IT and IG precipitates were not alloy carbides. It was also deduced that the precipitates in the ruptured pipe were most likely intermetallic compounds. The accumulation of Cr, Mo and W in the precipitates dispersed intergranularly and intragranularly might have contributed to the formation of an alloy-deficient zone adjacent to the precipitates. The chemical compositions of site E near the precipitates contained an obviously low amount of Mo and W, which implied the diffusion of Mo and W to the precipitates.

**Figure 4:**
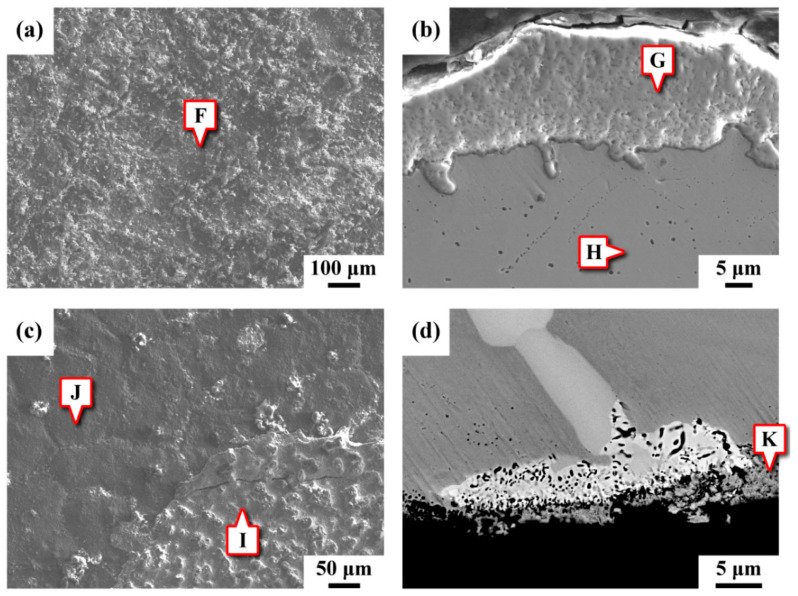
SEM micrographs showing the morphologies of the (**a**,**b**) external and (**c**,**d**) internal sections of the pipe in (**a**,**c**) top and (**b**,**d**) cross-sectional views.

Figure 4 presents SEM micrographs showing the morphologies of the external and internal sections of the pipe in top and cross-sectional views. The chemical compositions of the specific sites indicated in Figure 4 are provided in Table 3. The appearance of the external surface (Figure 4a) and cross-sectional microstructure near the free surface (Figure 4b) were examined by SEM. As shown in Figure 4b, the superficial scales comprised numerous pinholes. The chemical composition determined by EPMA (site F in Figure 4a, site G in Figure 4b) was associated with Cr oxide and consisted of very low Mo and W contents relative to the substrate. At the site 20 μm (site H) away from the interface between the oxide and substrate, the Cr content was low, implying that Cr diffused outward to form the Cr oxide layer. Figure 4c,d display the surface features and cross-sectional microstructure of the inner pipe, respectively. It seemed that there were two reactive products on the inner surface of the ruptured pipe (Figure 4c), i.e., a superficial covering (site I) and a subsurface (site J) covering. The subsurface zone vaguely displayed the grain boundary profiles. The corresponding chemical compositions, listed in Table 3, were applied to estimate the corrosion products formed on the inner surface of the pipe; the superficial covering (site I) was expected to be Ni oxide, and the subsurface one (site J) to be Mo-Cr oxide. Figure 4d shows the cross-sectional microstructure near the inner surface of the ruptured pipe. The grain boundary precipitates had the main composition of 38Mo-20Cr-5W in wt.% (not listed in Table 3), and this composition was similar to those of the large IG precipitates, listed in Table 2. The porous layer (site K) adhered to the inner surface of the pipe was a little lean in Cr, W and Mo but rich in Ni and O; such a surface covering was expected to be Ni oxide. The etched porous layer scaled off the pipe by the purged mist.

### 3.5. Crystal Structure Determination

**Figure 5:**
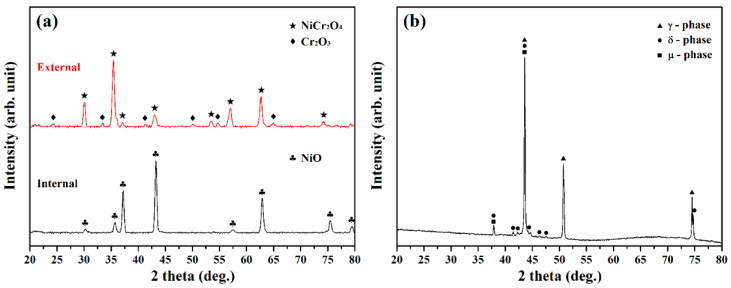
XRD patterns of the (**a**) external and internal surfaces and (**b**) substrate of the ruptured C-276 pipe.

The XRD patterns of the external and inner coverings and the ruptured pipe are displayed in Figure 5. As shown in Figure 5a, the surface covering consisted of NiCr_2_O_4_ mixed with Cr_2_O_3_, which was consistent with EPMA measurements (site F and G in Figure 4a,b). As shown in Figure 4c, the reactive products on the inner surface of the pipe were composed of two kinds of oxides. The chemical composition of site I in Figure 4c, which was enriched in Ni and O was related to NiO (Figure 5a). Based on the chemical analysis of site J in Figure 4c, it seemed that a Mo-Cr oxide layer had formed beneath the Ni oxide layer. Grinding and polishing were employed to form a flat area, and the XRD spectrum of the substrate of the inner pipe is shown in Figure 5b, revealing that two kinds of precipitates (δ and μ phases) were present in the γ matrix.

### 3.6. Results of Phase and Elemental Mapping

**Figure 6:**
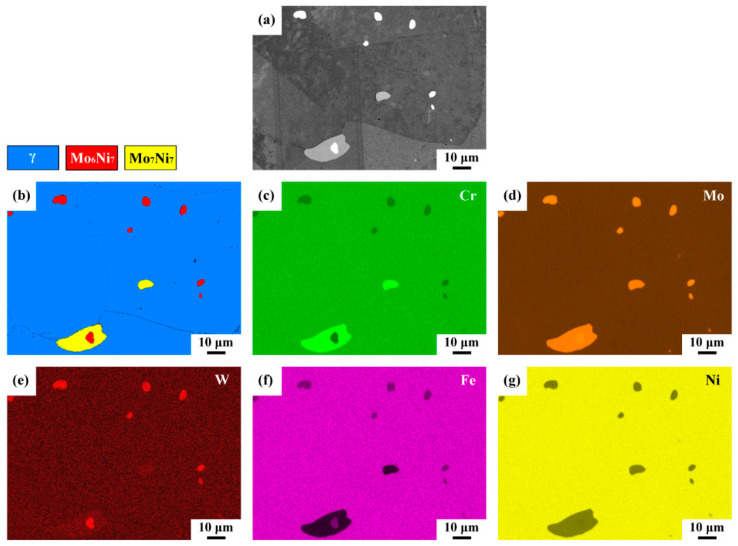
(**a**) SEM-BSE image and (**b**) EBSD phase map of the examined sample; (**c**–**g**) the compositional maps determined by EDS.

After metallographic preparations, the morphologies of the precipitates in the ruptured pipe were inspected by SEM in BSE mode, as shown in Figure 6a. Moreover, the precipitates in the sample were identified by EBSD phase map (Figure 6b). The distributions of the alloy elements in the examined sample were determined by energy-dispersive X-ray spectroscopy (EDS). Compositional maps (Figure 6c–g) were applied to confirm the EBSD analysis. The SEM-BSE image displays a few fine bright particles dispersed in the γ matrix and bright precipitates embedded in a large gray precipitate at the grain boundary (Figure 6a). The EBSD map confirmed that the fine particles were Mo_6_Ni_7_ (μ) and the large precipitate was Mo_7_Ni_7_ (δ) in the γ matrix (Figure 6b). As listed in Table 2, the μ and δ phases had similar Mo contents, but the μ phase was rich in W and lean in Cr relative to the δ phase. The compositional maps showed the differences in alloy concentrations in the μ, δ and γ matrix. These maps allowed the elemental distributions to be distinguished but lacked the accuracy of specific site measurement by EPMA. Overall, the maps demonstrated that the precipitates were lean in Fe (Figure 6f) and Ni (Figure 6g) relative to the substrate but enriched in Mo (Figure 6d). Therefore, the EDS mappings and EPMA analysis listed in Table 2 were consistent; the W tended to segregate to the μ phase (Figure 6e) but the δ contained a higher Cr concentration (Figure 6c).

### 3.7. Results of TEM Analysis

**Figure 7:**
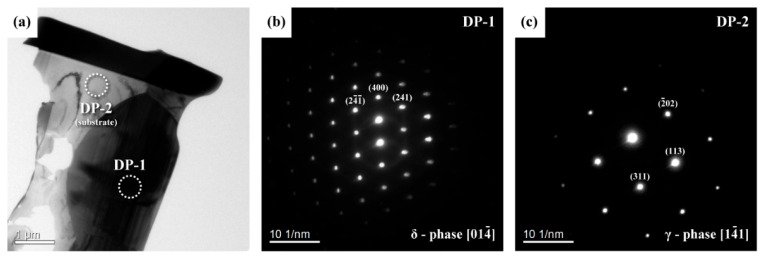
(**a**) Bright field TEM image of the FIB sample and the SADP of the (**b**) δ-phase and (**c**) substrate.

A bright field (BF) TEM micrograph and the selected area diffraction patterns (SADP) of the coarse precipitates at the grain boundaries, which were cut by FIB from the C-276 sample, are shown in Figure 7. SADP (Figure 7b) was used to identify the crystal structure of the precipitate. As reported in prior studies [17,18,19], the δ phase has an orthorhombic structure, and its lattice constants are a = b = 0.9108 nm, c = 0.8852 nm. The calculated lattice constants of the inspected precipitate were a = 0.9128 nm, b = 0.9134 nm, and c = 0.8836 nm, which were very close to the data disclosed in the open literature. Thus, the investigated coarse precipitate in the γ matrix was likely the Mo_3_(Mo_0.8_Ni_0.2_)_5_Ni_6_ [19] or Mo_7_Ni_7_ (δ) phase.

### 3.8. SEM Fractography

**Figure 8:**
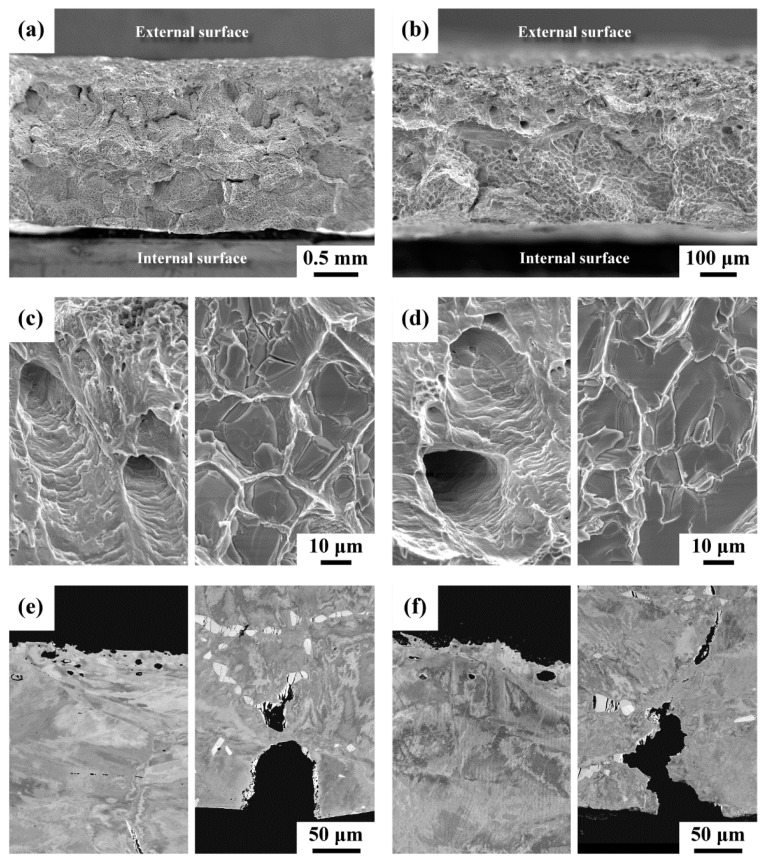
SEM micrographs showing the tensile fracture morphology and microstructure of the (**a**,**c**,**e**) thick sample and (**b**,**d**,**f**) thin sample: (**a**,**b**) the macro-fracture appearance; (**c**,**d**) the micro-fracture feature; (**e**,**f**) the microstructure around the tensile fracture zone. The left-hand image in (**c**–**f**) photos from around the outer surface and right-hand image in (**c**–**f**) photos from around the inner surface.

SEM micrographs of typical fracture morphology and microstructure around the outer and inner surfaces of the tensile samples are shown in Figure 8. Despite the thickness of tensile samples, the macro-appearance of the fractured sample displayed secondary crack growth along the coarse grain boundaries (Figure 8a,b). The occurrence of intergranular fractures around the inner surface of the pipe should be associated with numerous precipitates at the grain boundaries. In contrast, many fine pores were very likely found around the outer surface of the fractured pipe. Examining the surface features of the two fractured samples in Figure 8a,b at higher magnifications, tear-shear ruptures with deep holes were seen in the region around the outer surface (left-hand image in Figure 8c,d), which could be attributed to the porous structure around the external surface. Brittle flat facets were observed in the region around the inner surface (right-hand image in Figure 8c,d), which were associated with the separation along the grain boundary precipitates. To correlate the tensile fracture feature with the microstructure around the outer and inner surfaces of the pipe, a few samples were cut from the fractured tensile samples and subjected to metallographic preparations. The microstructure adjacent to the tensile fractured zone of the tested samples is shown in Figure 8e,f. Despite the original sample thickness, fine pores without any precipitates were observed in the outer surface zone (left-hand image in Figure 8e,f). Moreover, crack growth along the grain boundary and cracking of the coarse precipitates were observed in the inner surface region (right-hand image in Figure 8e,f). The higher extent of the etched layer and crack-extension zone in the thinner samples caused a higher degree of degradation in tensile properties than those of the thicker ones.

## 4. Discussion

Several different kinds of precipitates can be present in thermally aged C-276 alloy, depending on the aging conditions. It is reported that L + σ → P transformation occurs at above 1200 °C in Ni-Fe-Mo alloy [20]. The P and μ phases can form in the C-276 alloy when it is exposed to temperatures of 649 to 1093 °C [21]. With increasing aging time, the P phase will transform to μ phase [21]. In this work, SEM-BSE images and EBSD phase maps showed that the fine bright particles dispersed in the γ matrix were Mo_6_Ni_7_ (μ) phase. As listed in Table 2, the EPMA analysis elucidated that the bright fine particles were highly enriched in Mo and W but lean in Cr (38Mo-24W-7Cr in wt.%) relative to the substrate, which was consistent with prior results [15]. Alloy carbides have also been reported to be found in aged C-276 alloy [2,17]. The most abundant μ phase, the second most abundant M_6_C carbide and a few P phases were found in C-276 alloy aged in the temperature range of 650 to 900 °C [2]. However, alloy carbides were not found in this work, possibly due to the inherently low carbon content (0.003 wt.%) of this C-276 alloy. 

Coarse precipitates distributed in the γ matrix and along the grain boundaries were also inspected. Those coarse precipitates were rich in Mo and Cr, as listed in Table 2. The EBSD phase map and TEM inspection confirmed that they were Mo_7_Ni_7_ (δ) phase. The XRD pattern revealed the coexistence of δ and μ phase in the γ matrix. The difference in the chemical compositions of the two precipitates was that the W tended to segregate to the μ phase but the δ contained a higher Cr concentration. Because the μ phase contained much more W than did the δ phase, the SEM-BSE image of the former was brighter than that of the latter. It was also reported that the P phase has the same crystal structure as the δ phase [22]. Figure 3 shows the precipitation of δ phase (Cr-rich) and μ phase (W-rich) in the ruptured C-276 pipe. It was deduced that the accumulation of Cr and Mo enhanced the formation of δ phase in the C-276 pipe serviced at elevated temperature. The diffusivity of W in the C-276 alloy is much lower than that of Cr. After a certain time of service at elevated temperature, the aggregation of W assisted the transformation of δ to μ phase. Therefore, some of the fine μ phase were found to be embedded in the coarse δ in the ruptured pipe. Moreover, numerous precipitates dispersed in the matrix and along the grain boundaries caused an obvious degradation in tensile properties of the used C-276 pipe.

Dual oxide scales consisting of an outer NiO layer and an inner Cr_2_O_3_/NiCr_2_O_4_ mixed layer developed for C-276 alloy exposed in supercritical water at 500–600 °C/25 MPa [20,21], and some NiO reacts with Cr_2_O_3_ to form NiCr_2_O_4_ [23]. In this study, the outer surface of the pipe exposed to the electrical heater was covered by NiCr_2_O_4_ mixed with Cr_2_O_3_, whereas the reactive products on the inner surface of the ruptured pipe were identified as NiO. It is reported that the higher diffusivity of Ni than that of Cr contributes to the formation of the outer NiO layer in a Ni-based alloy [24]. As compared with the porous NiO [23], the compact Cr_2_O_3_/NiCr_2_O_4_ mixed layer can effectively protect the C-276 alloy in supercritical water [23]. The exact surface temperature of the inner pipe was unknown in this work. It was obvious that the formation of a porous NiO layer (Figure 4c,d) had less resistance to environmental corrosion and cracking. As shown in Figure 4b, many pin-holes and the absence of observable precipitates were noted beneath the surface oxide layer. The chemical composition at site H was obviously lean in Cr relative to the substrate. It was deduced that a large amount of Cr diffused out and reacted with O to form Cr_2_O_3_ and NiCr_2_O_4_ layers, leaving many pin-holes in the substrate adjacent to the oxide/metal interface.

The ruptured C-276 pipe was found to consist of many fine cracks around the hole and extending into the surrounding substrate and displayed a non-uniform reduction in thickness around the pipe. The reaction products of the decomposed perfluorinated compounds and the oxidizing environment in the pipe were the reasons for severe corrosion of the pipe. After carefully examining the ruptured pipe, the large amounts of IG precipitates were corroded preferentially, resulting in the formation of fine pores within the coarse precipitates. Moreover, the periodical purge with water mist introduced a temperature gradient between the outer and inner regions of the pipe, which inevitably induced high thermal stress. Therefore, thermal stress assisted the severe cracking of the grain boundary precipitates, resulting in the formation of microcracks along the grain boundaries. The coalescence of micro-pores into micro-cracks and the linkage of micro-cracks resulted in the fast crack growth along the grain boundaries of the pipe. Furthermore, the combination of thermal stress and corrosion attack caused spalling of the corroded segments, which was responsible for the rapid thinning in the wall of the used C-276 pipe. 

## 5. Conclusions

The causes of the rupture of a C-276 pipe used in a controlled decomposition reactor for catching the perfluorinated compounds in the exhaust gas of a foundry were investigated. The main findings are as follows:

1. The C-276 pipe used in a CDR system to catch decomposed perfluorinated compounds was etched to uneven thickness. The zone near the external surface had a porous structure, whereas the microcracks tended to initiate and grow from the inner surface of the pipe. The induced thermal stress in the pipe caused severe cracking of the grain boundary precipitates. Moreover, the grain boundary precipitates were corroded preferentially, resulting in the formation of fine pores within the precipitates and around them. The spalling of the corroded segments was responsible for the rapid thinning in the wall of the C-276 pipe.
2. Two kinds of precipitates were present in the ruptured C-276 pipe; the intergranular (IG) precipitates were coarser and greater in amount than the intragranular (IT) precipitates. The coarse precipitates at the grain boundaries were Mo_7_Ni_7_ (δ) phase, which was rich in Mo and Cr. By contrast, the fine precipitates dispersed intragranularly or embedded in the δ phase were the μ phase (Mo_6_Ni_7_) enriched in Mo and W.
3. After service at elevated temperature, the aggregation of W assisted the transformation of δ to μ phase in the aged C-276 pipe. Therefore, fine μ precipitates were found to be embedded in the coarse δ in the ruptured pipe. Moreover, numerous precipitates present in the matrix and along the grain boundaries caused a significant loss in the strength and ductility of the C-276 pipe.

## Figures and Tables

**Table 1:** Tensile properties of the tested samples cut from the used C-276 pipe *.

Sample	Thickness (mm)	Yield Strength (MPa)	Ultimate Tensile Strength (MPa)	Elongation (%)
A1	2.55	297	550	27
A2	2.52	281	540	31
A3	2.51	290	577	35
B1	0.85	271	505	11
B2	0.71	266	454	5
B3	0.66	252	452	4

* Note: The tensile properties of the as-received C-276 plate: yield strength 370 MPa, ultimate tensile strength 782 MPa, elongation 56%.

**Table 2:** Chemical compositions of sites A–E determined by EPMA in Figure 3.

Element	Site A		Site B		Site C		Site D		Site E	
	wt.%	at. %	wt.%	at. %	wt.%	at. %	wt.%	at. %	wt.%	at. %
**C**	0.1	1.0	0.1	0.8	0.1	1.0	0.2	1.0	0.2	1.0
**O**	0.0	0.1	0.0	0.0	0.0	0.0	0.0	0.0	0.0	0.0
**Cr**	7.1	12.1	21.4	30.3	7.0	12.3	21.5	30.3	13.2	15.8
**Mo**	37.9	35.1	36.0	27.7	34.2	32.5	36.7	28.0	9.0	5.8
**W**	23.6	11.4	4.5	1.8	26.4	13.1	4.9	2.0	1.8	0.5
**Fe**	2.1	3.3	1.2	1.6	2.0	3.3	1.1	1.5	6.7	7.4
**Ni**	bal.	bal.	bal.	bal.	bal.	bal.	bal.	bal.	bal.	bal.

**Table 3:** Chemical compositions of sites F–K determined by EPMA in Figure 4.

Element	Site F		Site G		Site H		Site I		Site J		Site K	
	wt.%	at. %	wt.%	at. %	wt.%	at. %	wt.%	at. %	wt.%	at. %	wt.%	at. %
**C**	0.9	2.6	0.5	1.3	0.2	1.0	1.6	5.5	2.4	7.4	0.4	1.4
**O**	26.4	55.0	37.2	68.5	0.0	0.0	12.8	33.5	24.4	56.8	9.1	28.5
**Cr**	51.4	32.9	58.3	30.1	7.1	8.7	6.9	5.5	9.5	6.8	11.6	11.2
**Mo**	9.1	3.2	0.0	0.0	18.2	12.1	0.4	0.2	38.6	15.0	14.2	7.4
**W**	1.6	0.3	0.1	0.0	3.7	1.3	1.7	0.1	4.6	0.9	3.0	0.8
**Fe**	1.9	1.1	0.1	0.0	6.2	7.1	2.8	2.1	1.9	1.1	0.4	0.3
**Ni**	bal.	bal.	bal.	bal.	bal.	bal.	bal.	bal.	bal.	bal.	bal.	bal.

## Data Availability

Not applicable.

## References

[1] Tawancy H.M., Herchenroeder R.B., Asphahani A.I. (1983). High-Performance Ni-Cr-Mo-W Alloys. JOM.

[2] Raghavan M., Berkowitz B.J., Scanlon J.C. (1982). Electron Microscopic Analysis of Heterogeneous Precipitates in Hastelloy C-276. Metall. Trans. A.

[3] Tawancy H.M. (1996). Precipitation characteristics of μ-phase in wrought nickel-base alloys and its effect on their propert. J. Mater. Sci..

[4] Akhter J.I., Shaikh M.A., Ahmad M., Iqbal M., Shoaib K.A., Ahmad W. (2001). Effect of aging on the hardness and impact properties of Hastelloy C-276. J. Mater. Sci. Lett..

[5] Zhao K., Ma Y.H., Lou L.H., Hu Z.Q. (2005). μ phase in a nickel base directionally solidified alloy. Mater. Trans..

[6] Zhang C., Zhang L., Cui Y., Feng Q., Cheng C. (2020). Effects of High-Temperature Aging on Precipitation and Corrosion Behavior of a Ni-Cr-Mo-Based Hastelloy C276 Superalloy. J. Mater. Eng. Perform..

[7] Tsai W.-T., Chen H.-P., Hsien W.-Y. (2002). A review of uses, environmental hazards and recovery/recycle technologies of perfluorocarbons (PFCs) emissions from the semiconductor manufacturing processes. J. Loss Prev. Process Ind..

[8] Lin A.Y.-C., Panchangam S.C., Lo C.-C. (2009). The impact of semiconductor, electronics and optoelectronic industries on downstream perfluorinated chemical contamination in Taiwanese rivers. Environ. Pollut..

[9] Golja B., Barkanic J.A., Hoff A. (1985). A review of nitrogen trifluoride for dry etching in microelectronics processing. Microelectron. J..

[10] Tsai W.-T. (2008). Environmental and health risk analysis of nitrogen trifluoride (NF3), a toxic and potent greenhouse gas. J. Hazard. Mater..

[11] Weiss R.F., Mühle J., Salameh P.K., Harth C.M. (2008). Nitrogen trifluoride in the global atmosphere. Geophys. Res. Lett..

[12] Robson J.I., Gohar L.K., Hurley M.D., Shine K.P., Wallington T.J. (2006). Revised IR spectrum, radiative efficiency and global warming potential of nitrogen trifluoride. Geophys. Res. Lett..

[13] Bakkar A., Ahmed M.M.Z., Alsaleh N.A., Seleman M.M.E.-S., Ataya S. (2019). Microstructure, wear, and corrosion characterization of high TiC content Inconel 625 matrix composites. J. Mater. Res. Technol..

[14] Manikandan M., Arivazhagan N., Nageswara Rao M., Madhusudhan Reddy G. (2015). Improvement of Microstructure and Mechanical Behavior of Gas Tungsten Arc Weldments of Alloy C-276 by Current Pulsing. Acta Metall. Sin..

[15] Ahmad M., Akhter J.I., Akhtar M., Iqbal M., Ahmed E., Choudhry M.A. (2005). Microstructure and hardness studies of the electron beam welded zone of Hastelloy C-276. J. Alloys Compd..

[16] Manikandan M., Hari P., Vishnu G., Arivarasu M., Ramkumar K.D., Arivazhagan N., Rao M.N., Reddy G. (2014). Investigation of microstructure and mechanical properties of super alloy C-276 by continuous Nd: YAG laser welding. Procedia Mater. Sci..

[17] Gozlan E., Bamberger M., Dirnfeld S.F., Prinz B., Klodt J. (1991). Topologically close-packed precipitations and phase diagrams of Ni-Mo-Cr and Ni-Mo-Fe and of Ni-Mo-Fe with constant additions of chromium. Mater. Sci. Eng. A.

[18] Shoemaker C.B., Shoemaker D.P. (1963). The crystal structure of the δ phase Mo-Ni. Acta Crystallogr..

[19] Nikolaev S.V., Kerimov E.Y., Slyusarenko E.M. (2014). Phase equilibria in four-component system Ni-Nb-Mo-Re at 1375 K. Inorg. Mater. Appl. Res..

[20] Liu J., Zhang J., Lu Y., Li X., Li Z., Zhou X. (2013). Effect of long-term aging on microstructure and mechanical properties of alloy C276. Acta Metall. Sin..

[21] Leonard R.B. (2013). Thermal Stability of Hastelloy Alloy C-276. Corrosion.

[22] Matthew F. (2019). Updated Sublattice Models of Topologically Close Packed Phases with a Revised Phase Description of σ Phase. Master’s Thesis.

[23] Zhang Q., Tang R., Yin K., Luo X., Zhang L. (2009). Corrosion behavior of Hastelloy C-276 in supercritical water. Corros. Sci..

[24] Ren X., Sridharan K., Allen T.R. (2007). Corrosion Behavior of Alloys 625 and 718 in Supercritical Water. Corrosion.

